# Supplementary material for: Patterns of Intron Gain and Loss in Fungi
Source: PLoS Biol. 2004 Nov 30;2(12):e422. doi: 10.1371/journal.pbio.0020422 (PMC532390; doi:10.1371/journal.pbio.0020422)
Supplement: Table S1 — Also available at http://genes.mit.edu/NielsenEtAl/. (4.3 MB ZIP). [file pbio.0020422.st001.zip › NielsenEtAl/html/1396.html]

AN0636.1.NCU09757.1.MG03933.1.FG00960.1


```
 CLUSTAL W (1.82) Multiple Sequence Alignments - Introns Inserted


Sequence 1: AN0636.1	488 aa
Sequence 2: FG00960.1	501 aa
Sequence 3: NCU09757.1	534 aa
Sequence 4: MG03933.1	499 aa
Alignment Length: 541 aa
Number Identitical Residues: 333 aa
Alignment Score (without introns) 14289


MG03933.1 	-MRRTYNIA~MVSDFFFPQPG~GVESHIYQLST~KLIDRGHKVIIITHQYPGRTGVRYLT
NCU09757.1	-MAPTYRIA~MVCDFFFPQPG~GIESHIYQLST~KLIDRGHKVIIITHAYEGRTGVRYLT
FG00960.1 	MTRRTYNIA~MVSDFFFPQPG~GIESHIYQLAT~KLVDRGHKVIIITHAYDDRKGIRYLT
AN0636.1  	---MPYNIA2MVSDFFFPQPG1GVESHIYQLST0KLIDRGHKVIIITHAYKGRTGVRYLT
          	    .*.** **.******** *:*******:* **:*********** * .*.*:****

MG03933.1 	NGLKVYHVPFLVMYRSATFPTVFSFFPIFRNIVIREQIDIVHGHASLSSLCHEAILHGRT
NCU09757.1	NGLKVYHVPFLVIYRSATFPTVFSFFPVFRNIVLRERIDIVHGHASLSSLCHEAILHART
FG00960.1 	NGVKVYHVPFLVIYRAATFPTVFSFFPIFRNICIRERIEIVHGHGSLSSLCHEAILHART
AN0636.1  	NGLKVYHVPFLVIYRETTMPTVFSFFPIFRNIVIREQIQIVHGHQSLSSFCHEAILHART
          	**:*********:** :*:********:**** :**:*:***** ****:*******.**

MG03933.1 	MGLRTVFTDHSLFGFADPASILTNKLLKFTLSDVDHVVCVSHTC~KENTVLRASLDPLMV
NCU09757.1	MGLRTVFTDHSLFGFADAASILTNKLLKFALSDVDHVICVSHTC2KENTVLRASLDPLMV
FG00960.1 	MGLRTVFTDHSLFGFADAGTILTNKLMKFILSDVDHSICVSHTC2KENTVLRASLDPVMV
AN0636.1  	MGLRTAFTDHSLFGFADAGSILTNKLLKFTLSDVDHVICVSHTC2KENTVLRASLDPLMV
          	*****.***********..:******:** ****** :****** ************:**

MG03933.1 	SVVPNAVVAENFRPVNY-----TAAGEQTTPPP--PP-----------------GPHDMI
NCU09757.1	SVIPNAVVADNFRPLGYPTTTSTPPSSISSPIPGLPPNPSSSLSSSLTPPRRILGPHDPI
FG00960.1 	SVIPNAVVAENFRPKDVPALPSPLGTSFNTEAPLYPP-------------PQRIGPRDTI
AN0636.1  	SVIPNAVVAENFRPLHATARANERQS--GGGSQIQPP-------------PRPIGPNDII
          	**:******:****   .: ..             **                 **.* *

MG03933.1 	TIVVISRLFYNKGTDLLTAAIPRILGSHPNTRFIIAGDGPKSIDLEQMIEQNVLQDRVEM
NCU09757.1	TIVVISRLFYNKGTDLLTAAIPRILAAHPNTRFIIAGSGPKSIDLEQMIEQNVLQDRVEM
FG00960.1 	TIVVISRLFYNKGTDLLIASIPRVLENHPNTRFIIAGSGPKAIDLEQMIETNVLQDRVEM
AN0636.1  	TIVVISRLFYNKGTDLLIAAIPRILASHPNVRFIIAGSGPKAIDLEQMLERNVLQDKVEM
          	***************** *:***:*  ***.******.***:******:* *****:***

MG03933.1 	LGPVRHEDVRNVMIRGQIYLHPSLTEAFGTVIVEAASCGLYVVCTQVGGIPEVLPSHMTV
NCU09757.1	LGPIRHSEVRDVMVRGHIYLHPSLTEAFGTVIVEAASCGLYVVCTQVGGIPEVLPGHMTV
FG00960.1 	LGPIRHEEVRDVMVRGHIYLHPSLTEAFGTVIVEAASCGLYVVCTAVGGIPEVLPSHMTT
AN0636.1  	LGAIRHEEVRDVMVRGHIYLHPSLTEAFGTVIVEAASCGLYVVCTRVGGIPEVLPQHMTT
          	**.:**.:**:**:**:**************************** ********* ***.

MG03933.1 	FARPDEDDLVAATGRAIAALRSGRVRTELFHEQVKTMYSWKNVAMRTERVYDGISGAISE
NCU09757.1	FAKPEEDDLVAATGKAIAALRRNEVRTELFHEQVKNMYSWTNVAERTERVYNGISGAISE
FG00960.1 	FAKPEEDDVVLATSKAISAIRAGKIRTEKFHEQVKKMYSWQNVAMRTERVYDGISGTISE
AN0636.1  	FAKPEEDDIVLATSKAISALRSNKVRTERFHDQVKVMYSWTDVARRTERVYKGISGDISP
          	**:*:***:* **.:**:*:* ..:*** **:*** **** :** ******.**** ** 

MG03933.1 	AEFYGYD-----------------GPAGGGPGLPSFALIDRLKRYYGCGIWAGKLFCLCV
NCU09757.1	AEFYGYDHLKDHRNGSNGSWTAVTATRGMRSGVQSFALIDRLKRYYGCGVWAGKLFCVCV
FG00960.1 	DEFYG------------------VDTSGYGSRIRNFALIDRLKRYYGCGIWAGKLFCLCC
AN0636.1  	QEFYGYYP---------------GEIQEAGDRVRNFALIDRLKRYYGCGVWAGKLFCLCV
          	 ****                           : .**************:*******:* 

MG03933.1 	IVDYLIFLFLEFWFPRDQIDICPDWSRSHKRKALRGTSHTNGGGVSRQSTENREYDQGRI
NCU09757.1	IVDYLIFLFLELVFPRESIDICPDWPRKKKVVDTR----REGGGLEWGDVE--EYRRRSR
FG00960.1 	VVDYLFFLFLEWWFPRDNIDICPDWPRKRP---------ADDDASSKKAAHSNRSSTSQG
AN0636.1  	VIDFLLYTFLEMWFPRANIDIARSWPKKLN---------GIDGDTAK--PEKERSG----
          	::*:*:: ***  *** .***. .*.:.             ..       .. .      

MG03933.1 	KAHIA
NCU09757.1	PSRLR
FG00960.1 	ASKLE
AN0636.1  	-----
          	
```
